# Interactions between gut microbiota, plasma metabolome and brain function in the setting of a HIV cure trial

**DOI:** 10.3389/fcimb.2025.1629901

**Published:** 2025-08-20

**Authors:** Alessandra Borgognone, Anna Prats, Ashish Arunkumar Sharma, Ignacio Martinez-Zalacaín, Carles Soriano-Mas, Christian Brander, Bonaventura Clotet, José Moltó, Beatriz Mothe, Rafick-Pierre Sekaly, Roger Paredes, Jose A. Muñoz-Moreno

**Affiliations:** ^1^ IrsiCaixa, Badalona, Spain; ^2^ Fundació Lluita contra les Infeccions, Badalona, Spain; ^3^ Department of Infectious Diseases, Hospital Universitari Germans Trias i Pujol, Badalona, Spain; ^4^ Department of Pathology and Laboratory Medicine, School of Medicine, Emory University, Atlanta, GA, United States; ^5^ Radiology Department, Hospital Universitari de Bellvitge, L’Hospitalet de Llobregat, Spain; ^6^ Translational Imaging Biomarkers Group, Institut D’Investigació Biomèdica de Bellvitge (IDIBELL), L’Hospitalet de Llobregat, Spain; ^7^ Institut D’Investigació Biomèdica de Bellvitge (IDIBELL) - Hospital Universitari de Bellvitge, Feixa Llarga, L’Hospitalet de Llobregat, Spain; ^8^ Centro de Investigación Biomédica en Red - Salud Mental (CIBERSAM), Instituto de Salud Carlos III, Madrid, Spain; ^9^ Departament of Social Psychology and Quantitative Psychology, Institute of Neurosciences, Universitat de Barcelona, Barcelona, Spain; ^10^ Universitat de Vic, Vic, Spain; ^11^ Centro de Investigación Biomédica en Red – Enfermedades Infecciosas (CIBERINFEC), Instituto de Salud Carlos III, Madrid, Spain; ^12^ Institución Catalana de Investigación y Estudios Avanzados (ICREA), Barcelona, Spain; ^13^ Center for Global Health and Diseases, Case Western Reserve University, Cleveland, OH, United States; ^14^ Universitat Politècnica de Catalunya – BarcelonaTech, Terrassa, Spain; ^15^ Faculty of Psychology and Education Sciences, Universitat Oberta de Catalunya, Barcelona, Spain

**Keywords:** neurocognition, microbiome, metabolome, HIV, vaccine trial

## Abstract

**Background:**

The intestinal microbiota composition has been linked to neurocognitive impairment in people with HIV (PWH). However, the potential interplay of microbial species and related metabolites, particularly in the context of an HIV cure strategy remains underexplored. The BCN02 trial evaluated the impact of romidepsin (RMD), used as a HIV-1 latency reversing agent and with reported beneficial neurological effects, combined with the MVA.HIVconsv vaccine on virus control during 32-weeks of monitored antiretroviral treatment interruption (MAP) in early-treated HIV-infected individuals. Here, we analyzed longitudinal gut microbiome, plasma metabolome and brain functioning data to identify potential associations and novel putative biomarkers of HIV-associated neurocognitive disorders (HAND).

**Methods:**

Data from fecal shotgun metagenomics, plasma metabolome, cognitive (standardized neuropsychological test score covering 6 cognitive domains, NPZ-6), functional (neuropsychiatric symptoms) and neuroimaging assessments were obtained and evaluated in 18 participants before and after RMD administration, and at the study end (post-MAP follow-up) in the BCN02 trial.

**Results:**

Participants with neurocognitive impairment (Lower vs. Higher NPZ-6 score group) were enriched in bacterial species, including *Desulfovibrio desulfuricans*, *Sutterella wadsworthensis* and *Streptococcus thermophilus*, and showed higher 1,2-propanediol degradation microbial pathway levels, before RMD administration. A multi-omics profiling showed significant and positive correlations between these microbial features and lipid-related metabolic pathways, previously linked to neurological disorders (i.e., sphingolipid, ether lipid, and glycerophospholipid metabolism), in participants with neurocognitive impairment, before RMD administration. Three indices (microbial-, metabolite-based and combined) obtained from the discriminant features were assessed longitudinally, showing progressive similarities between NPZ-6 score groups over time. Furthermore, the three indices and related discriminant features correlated negatively with functional outcomes, such as quality of life and daily functioning, and positively with depression, stress and CNS-related symptoms before RMD administration, while these associations became less discernible at the subsequent timepoints.

**Conclusions:**

While the direct effect of the intervention on the observed shifts cannot be conclusively determined in this study settings, these findings strengthen the link between gut bacteria, related metabolites, and neurocognitive function in PWH, and provide an analytical framework for future validation studies aimed at discovering predictive biomarkers for neurocognitive impairment in PWH.

## Introduction

Neuropathological and clinical interest in microbiome-gut-brain has exponentially grown in recent years. In the context of HIV infection, current evidence suggests that gut-associated dysbiosis may contribute to the pathogenesis of HIV-associated neurocognitive disorders (HAND) (Aizhen Hu et al., 2024). As progressive HIV infection is linked to gut microbiota perturbations (Vujkovic-Cvijin et al., 2013), leading to inflammation and immune dysregulation, intestinal dysbiosis could explain, at least in part, HIV infection-associated neurocognitive impairment.

Previous studies, mostly using 16S sequencing-based studies, identified differences in the gut microbiota composition of people with HIV (PWH) and neurocognitive impairment, compared to those without neurocognitive impairment (Aizhen Hu et al., 2024). Although the current evidence still remains controversial. Specifically, lower abundance in butyrate-producing bacteria and higher in *Klebsiella* along with increased bile acids and bioactive lipids, decreased vitamin D, terpenoids, and resolvin D1 were reported in HIV-positive individuals with HAND (Dong et al., 2021). Another study in women with HIV found higher abundance of *Methanobrevibacter, Odoribacter, Pyramidobacter, Eubacterium, Ruminococcus*, and *Gemmiger*, and lower abundance of *Veillonella* associated with cognitive impairment (Hua et al., 2023). Also, reduction in alpha diversity and relative increases in the ratio of *Blautia* and *Clostridium* to *Lachnospira* were described in PWH suffering from distal neuropathic pain (Ellis et al., 2022). Similarly, lower alpha diversity was reported in individuals with HAND compared to individuals without HAND, although stratified analyses for comparable demographic covariates did not recapitulate such pattern (Zhang et al., 2019).

In addition, some interventions have demonstrated the potential of long-term improvement in neurocognitive functions and global inflammation status via gut microbiota modulation in HIV-positive individuals with neurocognitive impairment. Examples are probiotic supplementation (Ceccarelli et al., 2017), docosahexanoic acid supplementation (Dong et al., 2022), or cannabinoids administration (McDew-White et al., 2023). While previous evidence provides a framework for linking the gut-brain axis with cognitive functioning in HIV, studies exploring potential associations between gut microbes, related metabolic byproducts and neurocognitive impairment in a strategy targeting HIV eradication are still lacking to date. Moreover, to the best of our knowledge, no study has investigated possible implications of the functional potential of gut microbes at the species-level in HAND.

The BCN02 was a proof-of-concept vaccine trial (NCT02616874) that assessed the safety and efficacy of a kick&kill strategy, combining low-dose of the latency reversing agent (LRA) romidepsin (RMD) with a therapeutic HIV vaccine (MVA.HIVconsv), followed by a 32-week monitored antiretroviral pause (MAP) to evaluate virus control post-intervention in 15 early-treated HIV-1-positive individuals (Mothe et al., 2020). Romidepsin administration led to transient increases in histone acetylation, cell-associated HIV-1 RNA levels, and T-cell activation. During MAP, 23% of individuals showed sustained suppression of viremia up to 32 weeks without evidence for reseeding the viral reservoir.

A previously published BCN02 sub-study exploring longitudinal gut microbiota patterns associated with the main trial outcomes, suggested *Bacteroidales*/*Clostridiales* ratio as a potential microbial signature associated with HIV-1 reservoir size and immune-mediated viral control after ART interruption (Borgognone et al., 2022). In addition, the inclusion of RMD, previously described in neurofunction modulation and central nervous system (CNS) protection (Chan and Ananworanich, 2019; Zeng et al., 2021), provided the opportunity to evaluate its effect on virus reactivation and neurological impact in the BCN02 trial, including cognitive status, functional outcomes, and neuroimaging assessments in the trial participants (Munoz-Moreno et al., 2022).

In this exploratory study, we have characterized the intestinal microbiome and plasma metabolome profile in early treated HIV-1-positive individuals with different cognitive functioning in the BCN02 trial to assess potential interactions between specific microbial, metabolic patterns and neurocognitive impairment as well as the potential impact of a HIV therapeutic intervention, including the LRA romidepsin.

## Methods

### Study participants and design

**Figure 1:**
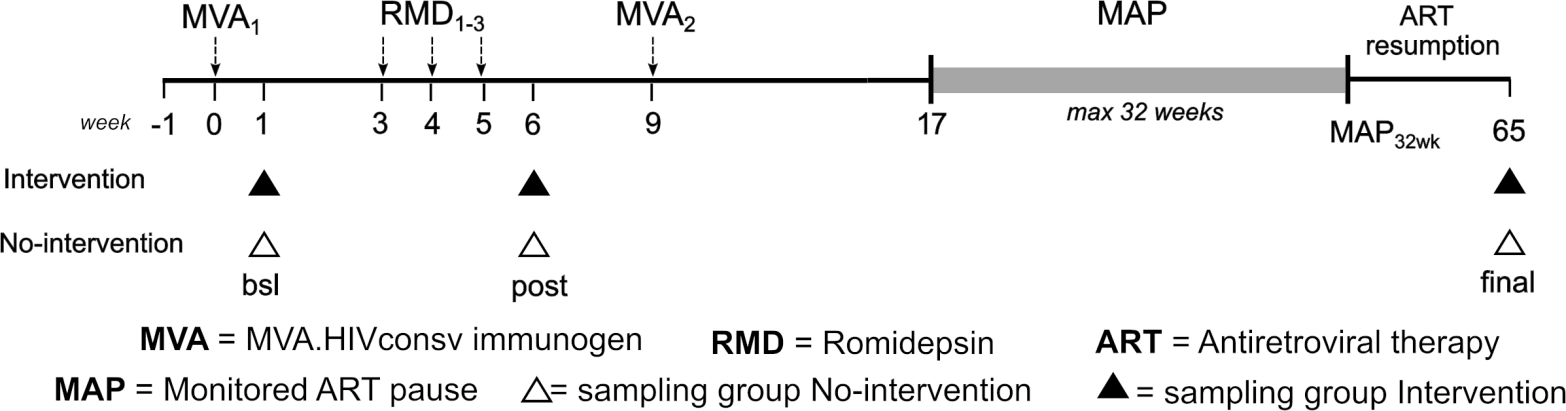
Study design and longitudinal sampling. bsl, baseline; post, post romidepsin administration; final, end of study.

The BCN02-CNS sub-study (Munoz-Moreno et al., 2022) investigated the effects of romidepsin combined with an HIV.consv vaccine on CNS in early-treated HIV-infected individuals (*Intervention* group) and compared these profiles to a group of early-treated HIV-infected subjects with equivalent clinical characteristics, not receiving the combined intervention and used as a control (*No-Intervention* group) (**Figure 1**).

Cognitive, functional and neuroimaging assessments were performed before (bsl), after RDM administration (post) and at post-MAP ART resumption follow-up (final), as detailed in (Munoz-Moreno et al., 2022). A global cognitive functioning index (http://links.lww.com/QAD/C388) based on the mean of the 6 standardized measures of cognitive domains (NPZ-6 score) was calculated and participants classified into Lower NPZ-6 and Higher NPZ-6 groups at the baseline (NPZ-6 score threshold = - 0.5).

Available CNS functioning and fecal shotgun metagenomics (Borgognone et al., 2022) data from participants with Lower (≤-0.5) and Higher (>-0.5) NPZ-6 score (**Supplementary Figure S1**) were crossed and used in this sub-study (Lower NPZ-6 score, n=3 and Higher NPZ-6 score, n=15). Characteristics of the study participants are shown in **Table 1** (sex-specific data points were highlighted in the plots, when applicable). Mediterranean diet adherence questionnaire (PREDIMED) data were available for the study participants, showing no significant differences between NPZ-6 score groups by comparing food categories and global adherence score.

**Table 1:** Characteristics of the study participants.

	Lower NPZ-6 (n=3)	Higher NPZ-6 (n=15)	P-value
Age (years)	39 (37.5 - 39.5)	40 (34 - 47)	0.592
Sex, n (%) (male)	2 (67)	15 (100)	0.021
Ethnicity, n (%) (caucasian)	2 (67)	14 (93)	0.180
Formal education (years)	12 (10.5 - 13.5)	15 (12 - 16.5)	0.231
CNS-related comorbidities	2 (67)	10 (67)	1.000
Time on cART (years)	3.2 (3.1 - 3.5)	3.3 (3.1 - 3.4)	0.811
Current cART regimen, n (%)			0.701
TDF/FTC/RAL	2 (11)	9 (50)	
ABC/3TC/RAL	1 (6)	2 (11)	
ABC/3TC/DTG	0 (0)	3 (17)	
TDF/FTC/EVG/Cob	0 (0)	1 (6)	
CD4+ T-cell counts (cells/mm3)	1331 (910 - 1369)	860 (652 - 1076)	0.497
CD4+/CD8+ ratio	1.2 (1.1 - 1.4)	1.3 (1.2 - 1.8)	0.737

Median (IQR) are shown unless otherwise indicated. Chi-square test was used for categorical variables. Mann–Whitney test was used when median is reported. 3TC, lamivudine; ABC, abacavir; Cob, cobicistat; DTG, dolutegravir; EVG, elvitegravir; FTC, emtricitabine; RAL, raltegravir; TDF, tenofovir.

### Fecal shotgun metagenomics sequencing and analysis

Total fecal DNA for gut microbiota profiling was extracted using the PowerSoil DNA Extraction Kit (MO BIO Laboratories, Carlsbad, CA, USA) and then cryopreserved at −80°C without preservation buffer until DNA extraction. Extracted DNA was fragmented using a Nextera-XT DNA Library Kit (Illumina, CA, USA) and one library of approximately 300-basepair- clone insert size constructed for each sample. Metagenomic sequencing libraries were processed on an Illumina Hi-Seq platform (Illumina, CA, USA) (expected approximately 20 million paired end sequences per sample). Sequence quality was assessed using the FastQC software (Andrews et al., 2015). FASTQ sequence files were filtered by length and quality using Trimmomatic (Bolger et al., 2014) ensuring a minimum base quality of Q30 for both leading and trailing bases, a minimum length of 75 basepairs and a minimum sequence quality average of Q20 for 30 basepair sliding windows across sequences. Filtered sequences were mapped against the human genome using Bowtie2 software (Langmead and Salzberg, 2012) to remove host DNA contamination. MetaPhlAn2 software (Truong et al., 2015) was used for species assignment and quantify the relative abundance at different taxonomy ranks. Filtered sequences were mapped against the Integrated Gene Catalog (IGC) (Li et al., 2014) using Bowtie2 and gene richness estimated as the total number of different genes present in the sample regardless of their abundance and length, as described previously (Le Chatelier et al., 2013). Metabolic pathways and gene families were determined using HUMAnN2 (Franzosa et al., 2018).

### Plasma metabolomics analysis

For host metabolome profiling, 200 μL frozen plasma were shipped to Metabolon, Inc (Morrisville, NC, United States). Plasma metabolite levels for 1300 biochemicals were measured using ultra-high-performance liquid chromatography-tandem mass spectrometry (UHPLC/MS/MS) on the Metabolon DiscoveryHD4^®^ platform. The data generated using the UHPLC/MS/MS were referenced against a well-established library of known and novel metabolites.

Discriminant metabolites contributing to the explanation of the most variance between comparison groups were identified based on a sparse Partial Least Squares Discriminant Analysis (sPLS-DA), followed by principal component analysis (PCA) for variable selection using the R package mixOmics (Rohart et al., 2017). Variables contributing to the two main components in the sPLS-DA model (N=100 per component) were sorted based on their loading weight and used as input data for pairwise comparison (Wilcoxon rank-sum test. *p* values < 0.05). The resulting discriminant metabolites were represented in a hierarchical clustering analysis (HCA), with Euclidean distance and Ward linkage as main parameters of the model (unnamed biochemicals were filtered out).

Pathway enrichment analysis of metabolic data (MSEA) on discriminant metabolites was performed using the MetaboAnalyst webserver v5.0 (Chong et al., 2019). KEGG human metabolic pathway database was set as reference metabolite library.

### Brain function assessment

Cognitive functioning was assessed using a comprehensive battery of neuropsychological tests that evaluated 6 cognitive domains, specifically attention/working memory, information processing speed, memory/learning, executive function, verbal fluency, and motor function. The six resulting measures were standardized to *z* scores. A final global *z* neuropsychological index was obtained calculating the mean of the six measures, which was the NPZ-6 score. Descriptive variables of the sample included cognitive complaints according to the European AIDS Clinical Society (EACS) proposal (EACS, 2019), as well as comorbidities previously described to potentially confound cognitive impairment according to the Frascati proposal (i.e. HCV co-infection, psychiatric disease, psychopharmacological treatment, drug use, and CNS-related disease) (Antinori et al., 2007).

Functional outcomes recorded were based on CNS-related symptoms, assessment of daily living functioning, emotional status, and quality of life. CNS-related symptoms were evaluated by an adapted FDA-based symptom checklist. Daily living functioning was evaluated by a Spanish version of the Interference of Activities of Daily Living (IADL) scale (Muñoz-Moreno et al., 2017). Emotional status assessment covered depressive and anxiety symptoms with the Hospital Anxiety and Depression Scale (HADS) (Zigmond, 1983) and perceived daily stress with the Perceived Stress Scale (PSS) (Cohen and Williamson, 1988). Quality of life was evaluated by an abbreviated version of the Medical Outcomes Study (MOS) - HIV questionnaire (Wu et al., 1991).

Neuroimaging data were collected in a 3 Tesla Magnetic Resonance Imaging (MRI) Siemens Verio scanner (Siemens Healthcare Sector, Erlangen, Germany). A high resolution T1-weighted 3-D structural image in the axial plane was obtained for each participant (thalamus and striatal, limbic and frontal cortex areas from left- and right-hemispheres), using the following parameters: 192 slices; repetition time = 1900 ms; echo time = 2.72 ms; flip angle = 9°, field of view = 260 × 260 mm; matrix size 256 × 256 pixels, in-plane resolution = 0.96 × 0.96 mm^2^; slice thickness = 0.9 mm.

### Statistical analysis

Significant differences in clinical characteristics were evaluated with Chi-square test. Gut microbiota profiling was performed using R/phyloseq (McMurdie and Holmes, 2013). All pairwise comparisons were performed using a two-sided Wilcoxon rank-sum test (Mann-Whitney U test). Multiple group comparisons were conducted using Kruskal-Wallis test. For beta diversity, statistical significance was assessed using the pairwise permutational multivariate analysis of variance (PERMANOVA) test (R/*adonis2* function) (Anderson, 2017). The linear discriminant analysis effect size (LEfSe) (Segata et al., 2011) was performed to identify discriminant bacterial signatures (α = 0.05 and LDA score > 2.0). Spearman’s rank correlations and BH-adjusted p-values (≤0.05) were calculated using the R/*rcorr* function.

Neuroimaging data were pre-processed and analyzed using MATLAB 7.14 (The MathWorks, Natick, MA, USA) and Statistical Parametric Mapping (SPM12; The Welcome Department of Imaging Neuroscience, London, UK). Longitudinal pre-processing consisted of an initial rigid-body within-subject co-registration to the first scan to ensure good starting estimates, followed by a pairwise longitudinal registration between the scans of each participant to obtain an average image and a Jacobian difference map. The average image was then segmented and the gray matter voxels were multiplied by the Jacobian difference map to obtain a subject-specific gray matter volume change map. Next, using a Diffeomorphic Anatomical Registration Through Exponentiated Lie (DARTEL) algebra algorithm, a template of our study sample in Montreal Neurological Institute (MNI) space was generated, which was used to spatially normalize the gray matter volume change maps. A one-way ANOVA approach within SPM was used to compare volume changes across the study groups. Significance threshold was set at p<0.05 family-wise error (FWE) corrected for multiple comparisons across the whole-brain voxels.

Data analysis was performed using R version 4.1.2.

## Results

### Baseline microbiota and metabolic patterns associated with neurocognitive impairment

Gut microbiota and plasma metabolome before romidepsin administration were firstly characterized to identify potential signatures related to distinct brain functioning.

**Figure 2:**
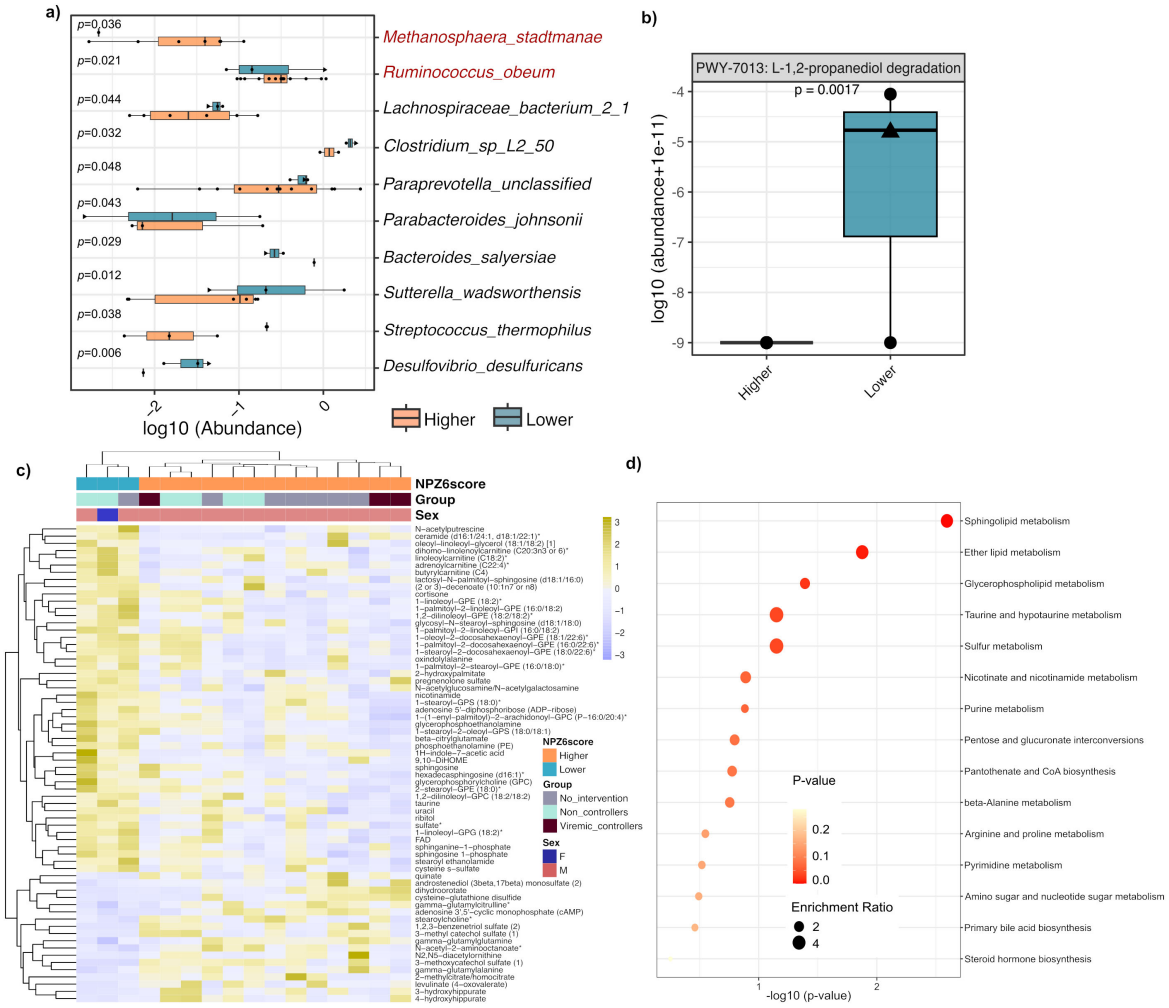
Discriminant gut bacteria and plasma metabolome features in BCN02 participants with different NPZ-6 score at baseline. **(a)** Differentially abundant bacterial species based on LEfSe algorithm (*p*<0.05). Bacteria increased in Higher and Lower NPZ6 groups are marked in red and black, respectively. **(b)** Comparison of the 1,2-propanediol degradation microbial metabolic pathway. **(c)** Heatmap clustering of differentially abundant plasma metabolites (Mann–Whitney–Wilcoxon test performed in the sPLS-DA output, *p*-value < 0.05) between Lower and Higher NPZ-6 groups. Intervention group as well as viral control during MAP are labeled. **(d)** Enrichment analysis of KEGG pathways from the metabolite set significantly increased in the Lower NPZ6 group. Male and female participants are indicated with a circle and triangle, respectively.

In the microbiome analysis, the species-level composition showed no global differences between Lower and Higher NPZ-6 groups (**Supplementary Figure S2A**). Diversity analysis showed that microbial gene richness (**Supplementary Figure S2B**) and beta diversity (Bray-Curtis distances) (**Supplementary Figure S2C**) were not significantly different between the two groups. In the linear discriminant analysis (LEfSe), the gut microbiota of participants with Lower NPZ-6 score was found enriched in bacterial species, including *Desulfovibrio desulfuricans, Sutterella wadsworthensis* and *Streptococcus termophilus* among others (**Figure 2a**). To improve the classification accuracy, a random forest prediction model was performed. Among the 10 top-ranked bacteria, 86% matched with the differentially abundant species identified by the LEfSe algorithm (**Supplementary Figure S3**). In the community-level comparison of microbial metabolic pathways (global mean > 1%), the gut microbiome of the Lower NPZ-6 group was functionally enriched in the 1,2-propanediol degradation pathway (**Figure 2b**), which is contributing to propionic acid synthesis (Metacyc DB) (**Supplementary Figure S4**).

In the metabolome analysis, the sPLS-DA plot based on the key discriminant metabolites differentiated between participants with Higher and Lower NPZ-6 score with no overlap (**Supplementary Figure S5** and **Supplementary Table S1**). Discriminant metabolites from the sPLS-DA followed by pairwise comparison (see Methods) (N=64, **Supplementary Table S2**) represented in a heatmap showed sample clustering based on the NPZ-6 score (**Figure 2c**). Of note, samples from Intervention and No-intervention groups (Intervention group stratified by viremic controllers and non-controllers, as in (10, 11) also tended to cluster based on differential metabolic features (**Figure 2c**). Enrichment analysis of KEGG pathways (64 discriminant metabolites used as input) by NPZ-6 score group revealed 5 (out of 15) pathways significantly enriched in Lower NPZ-6 group: sphingolipid metabolism (*p* = 0.000589, adjusted- *p* = 0.0377), ether lipid metabolism (*p* = 0.00672, adjusted- *p* = 0.0506), glycerophospholipid metabolism (*p* = 0.0188), taurine and hypotaurine metabolism (*p* = 0.0402) and sulfur metabolism (*p* = 0.0406) (**Figure 2d** and **Supplementary Table S3**). In contrast, only two, statistically not significantly enriched pathways were identified in the Higher NPZ-6 group (**Supplementary Figure S6** and **Supplementary Table S3**).

**Figure 3:**
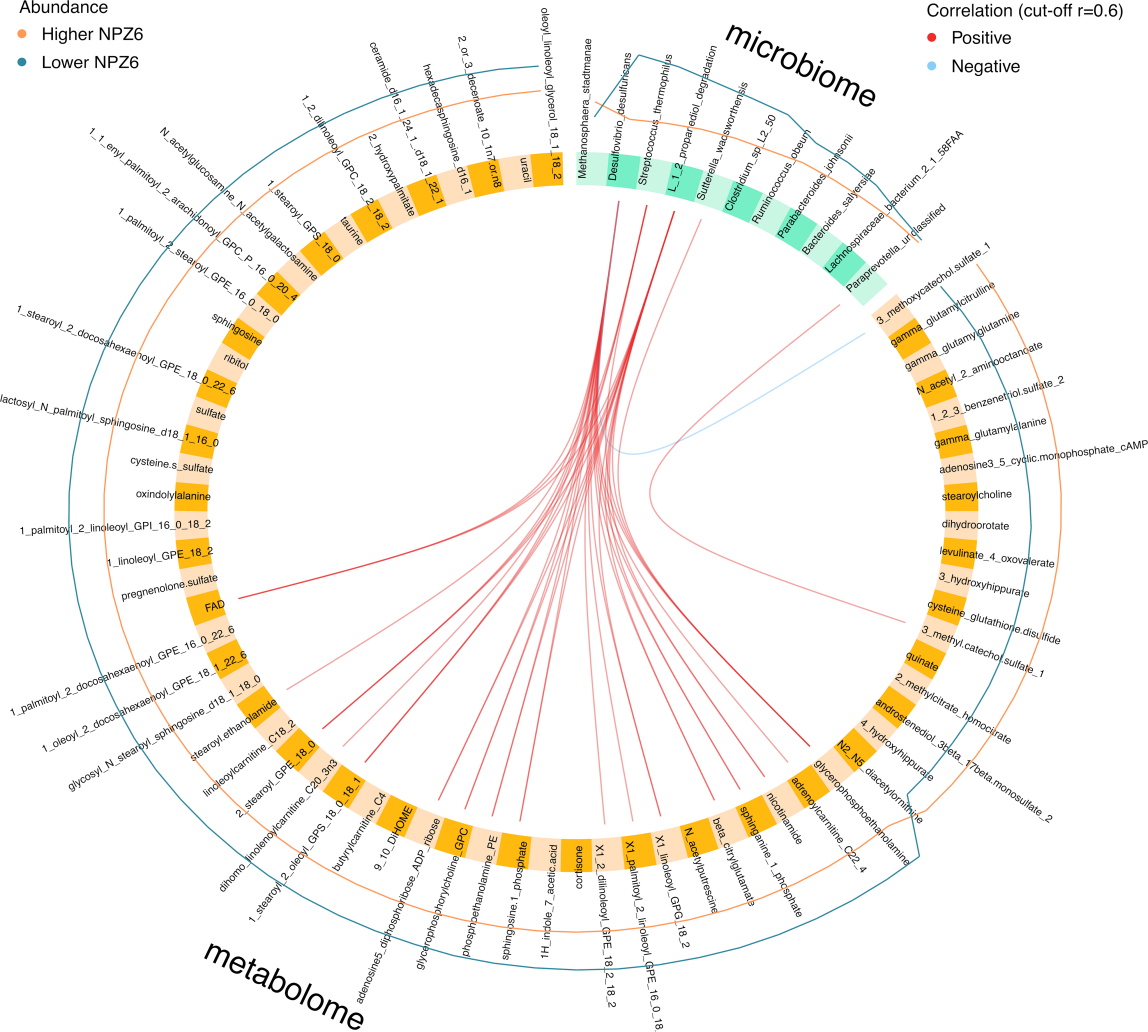
Integrated analysis of gut microbiome and plasma metabolome data. Circos plot representing correlations (cutoff r=0.6) between discriminant gut bacteria (microbiome block) and plasma metabolites (metabolome block). Inner red and light blue lines correspond to positive and negative correlations between connected features, respectively. Outer orange and dark blue lines indicate the variation of each feature in Higher and Lower NPZ6 groups, respectively.

In the integration analysis, we found positive correlations between discriminant microbial and metabolomic signatures increased in the Lower NPZ-6 group (**Figures 2a–c**), while only one negative correlation was observed between *D. desulfuricans* and the 3-methoxycatechol sulfate metabolite increased in the Higher NPZ-6 group (**Figure 3**). In particular, *D. desulfuricans, S. thermophilus, S. wadsworthensis* and the 1,2-propanediol degradation microbial pathway were positively associated with 17 metabolites, mostly involved in lipid biosynthesis and metabolism. Of note, using a more stringent correlation cut-off (r=0.7), only *D. desulfuricans* and the 1,2-propanediol degradation pathway showed positive correlations with 4 metabolites, also involved in lipid and phosphatidylethanolamine metabolism (glycerophosphoethanolamine, nicotinamide, 1-linoleoyl-GPG (18:2)* and phosphoethanolamine).

### Microbial and metabolome-based indices for distinct brain functioning

**Figure 4:**
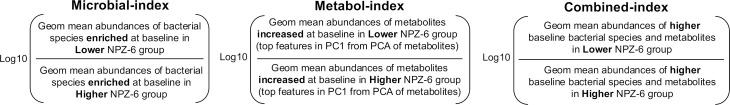
Design of microbial, metabolome and combined-based indices. Microbial, metabolome and combined-index calculation. features for each index are listed in **Supplementary Table S4**.

To further explore potential microbiota and metabolomic patterns associated to distinct brain functioning, we calculated a microbial-based and metabolome-based as well as a combined index, by collapsing the discriminant features between participants with Lower vs. Higher NPZ-6 score into a single variable. Briefly, we calculated the log ratio of geometric mean abundances of bacterial species enriched in the Lower NPZ-6 group (Wilcoxon *p-value* < 0.05) over geometric mean abundances of bacterial species enriched in Higher NPZ-6 group (Wilcoxon *p-value* < 0.05), at baseline. To build the metabolome-based index, we firstly performed a principal component analysis (**Supplementary Figure S7**) based on the differentially abundant metabolite set (**Figure 2c**). To then identify the most discriminant features, we extracted the top negative and positive components of the first principal component (PC1 contribution > 0.15 and < -0.10) and applied the same formula, by collapsing the top discriminant metabolites (the obtained features are listed in **Supplementary Table S4**). Finally, the combined-index was obtained by merging the bacteria and metabolites from numerator and denominator of the microbial and metabolome-based indices, respectively (**Figure 4** and **Supplementary Table S4**).

### Longitudinal dynamics of the predictive indices in cognition, functional and neuroimaging outcomes

**Figure 5:**
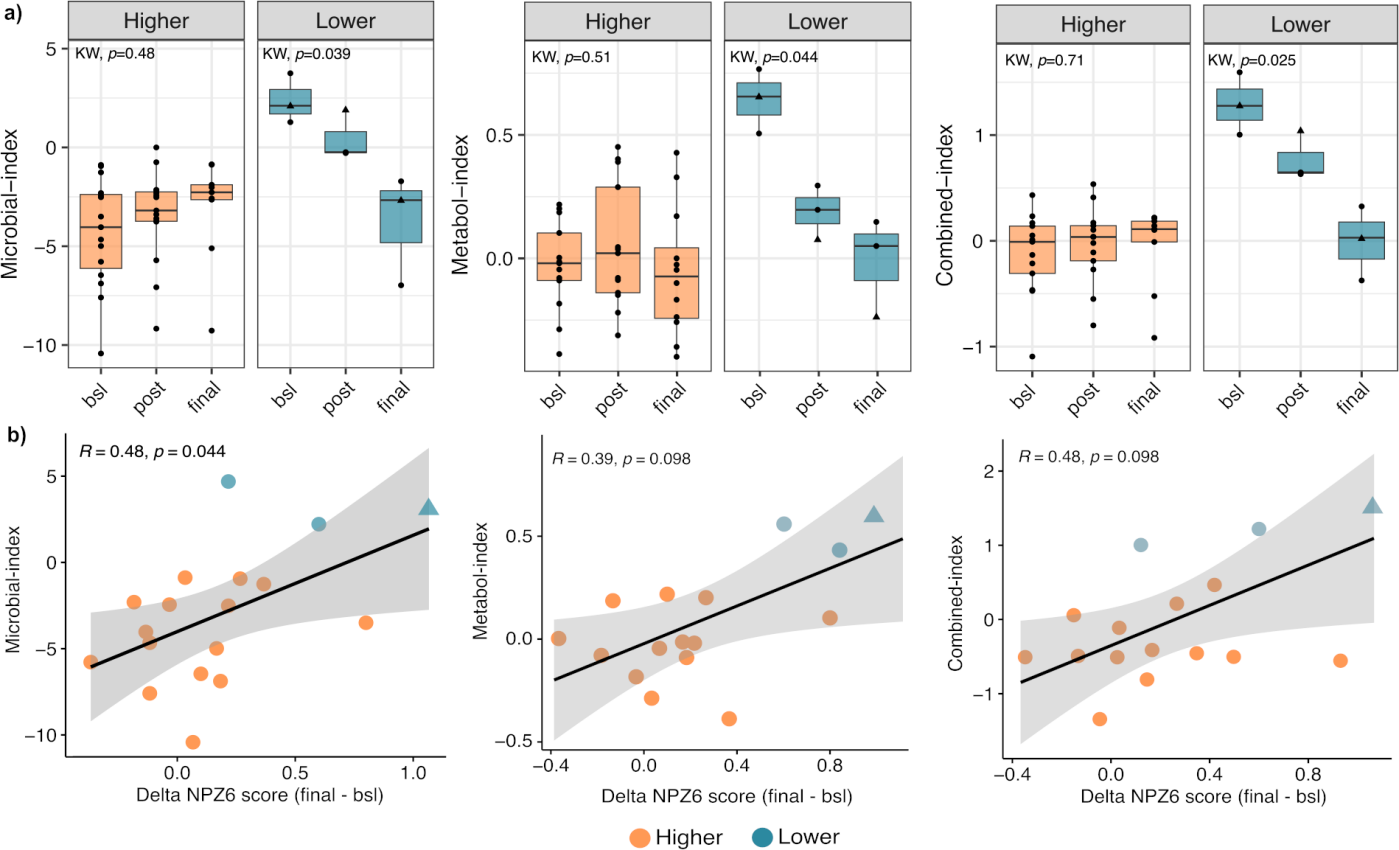
Temporal dynamics of the indices over the trial. **(a)** Longitudinal comparison of microbial, metabolome and combined-based indices between Lower and Higher NPZ-6 groups; **(b)** Spearman’s correlation analysis between the predictive indices and delta NPZ-6 score. Male and female participants are indicated with a circle and triangle, respectively.

We next evaluated the temporal dynamics of the predictive indices over the intervention of the BCN02 HIV clinical trial. In participants with Lower NPZ-6 score, all the three indices showed a significant decrease from baseline to the end of the trial (**Figure 5a**). Pairwise comparison within each index revealed significant differences before (Microbial, *p*=0.0025, Metabolome, *p*=0.0036 and Combined, *p*=0.0033) and after romidepsin administration (Microbial, *p*=0.0067, and Combined, *p*=0.011) but not at the study end between Lower and Higher NPZ-6 group. Indeed, at the end of the trial (final), the indices showed similar values in the two groups (**Figure 5a**) and positive correlation with delta NPZ-6 score (final – bsl) (**Figure 5b**).

**Figure 6:**
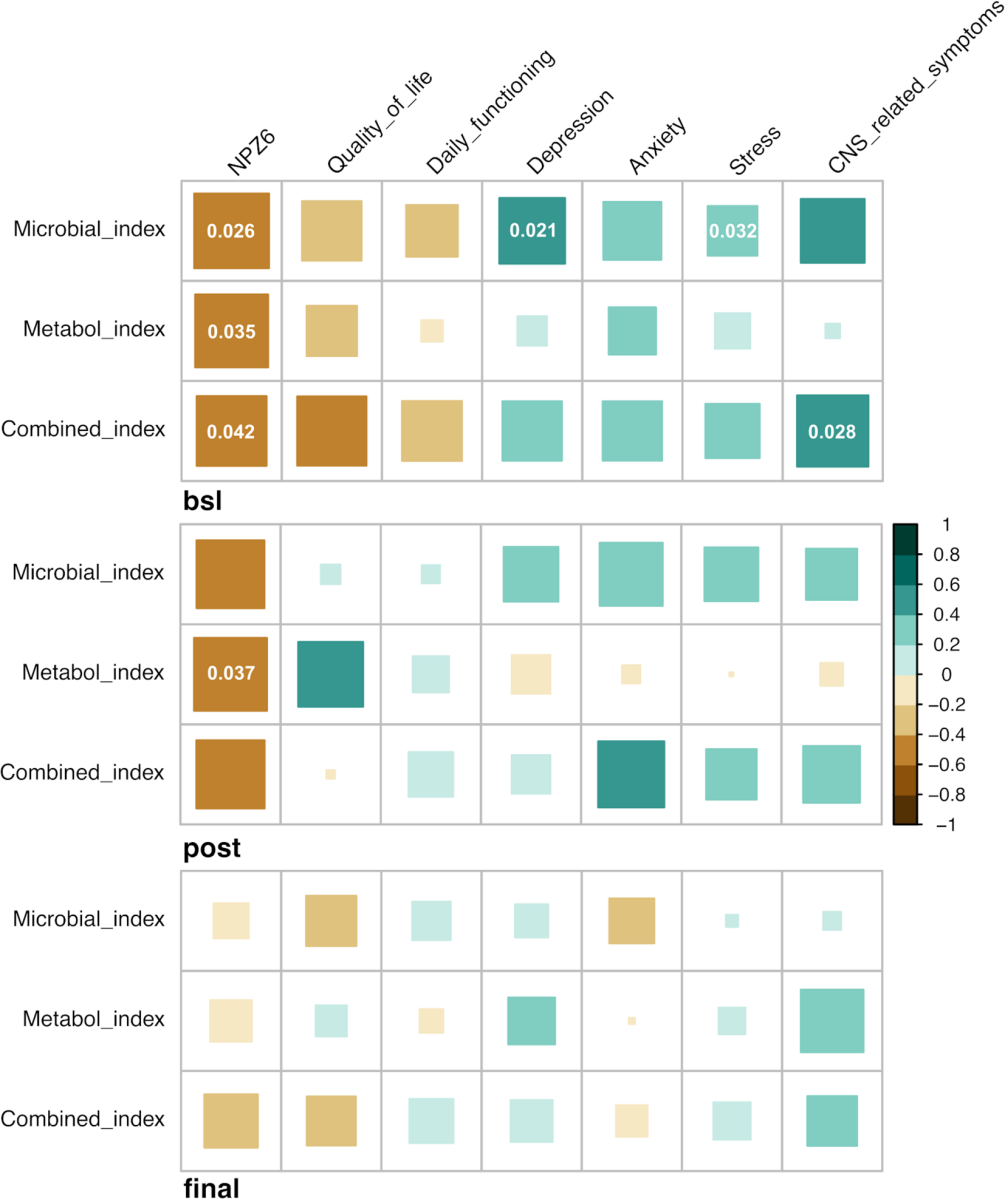
Associations between predictive indices, cognitive and functional outcomes. Heatmap of Spearman’s correlations between microbial, metabolome and combined-based indices and functional outcomes over the trial. Green and brown colors indicate positive and negative correlations, respectively. Correlations with statistical significance (*p < 0.05; Benjamini–Hochberg adjustment for multiple comparisons) are displayed.

In addition to the cognitive outcome, we sought to identify possible associations between the three estimated indices and functional outcomes over the trial. At baseline, we found that the three indices were significantly and negatively correlated with the NPZ-6 score, quality of life and daily functioning and positively with depression, stress and anxiety and CNS-related symptoms (**Figure 6**). After RMD administration, similar correlation patterns were found, although with less significance, whereas the observed patterns were less distinguishable at the study end (**Figure 6**). Also, to provide additional insights on the key features involved in the observed associations, we performed correlation analyses between the index-associated bacteria and metabolites and the cognitive and functional outcomes. Overall, at baseline, bacteria (i.e. *D. desulfuricans* and *S. wadsworthensis*) and metabolites (i.e. N-acetylglucosamine/N-acetylgalactosamine, glycerophosphorylcholine and sphingosine 1-phosphate) increased in the Lower NPZ-6 group were negatively correlated with NPZ-6 score, quality of life and daily functioning and positively with depression, stress and anxiety and CNS-related symptoms (**Supplementary Figure S8**), similarly to what was observed for the collapsed indices (**Figure 6**). Conversely, an opposite trend was observed for the features increased in the Higher NPZ-6 group (such as the *R. obeum*, *M. stadtmanae* bacteria and the 3-methoxycatechol sulfate, 1,2,3-benzenetriol sulfate metabolites) (**Supplementary Figure S8**). Consistent with the trend described in **Figure 6**, the observed patterns tended to lessen at post romidepsin and at the study end, although strong and significant correlations between a group of metabolites increased in Lower NPZ-6 group (including 1-stearoyl-2-oleoyl-GPS, 2-stearoyl-GPE, glycerophosphorylcholine, sphinganine-1-phosphate and phosphoethanolamine) and functional outcomes were found. Finally, to further understand possible implications in the CNS, we examined associations between neuroimaging measures and the predictive indices. In the longitudinal comparison of four brain regions (voxel-wise volumes), including the thalamus and striatal, limbic and frontal cortex areas (left- and right-hemispheres), no significant differences were found between Lower and Higher NPZ-6 groups (**Supplementary Figure S9**). Also, no significant correlations were identified between the indices and neuroimaging measures in any of the four brain regions, assessed before romidepsin administration (timepoint with strongest observed signatures in the previous determinations) (**Supplementary Figure S10**).

## Discussion

A number of studies have reported associations between gut microbiota alterations and neurocognitive impairment in PWH (Heaton et al., 2011; Sacktor et al., 2016). Here, we identified discriminant gut microbial and metabolic signatures associated with distinct brain functioning in PWH, before entering a HIV therapeutic vaccine trial. Such signatures were identified before the intervention while the potential impact of RMD administration on the observed changes over the trial remained unclear. Combining the discriminant features in a simple metric, we also proposed biologically interpretable indices to link microbial and metabolite patters with distinct cognitive and functional outcomes in this study setting.

In particular, the bacterial species enriched in participants with neurocognitive impairment at baseline before romidepsin administration and previously described in other neurocognitive disorders, such as *D. desulfuricans*, *S. wadsworthensis*, *S. thermophilus* and *Clostridium* spp., showed negative correlations (as single species and collapsed in the microbial-index) with functional outcomes, such as quality of life and daily functioning and positive with CNS related symptoms, depression, stress and anxiety. On the other hand, an opposite trend was observed for bacteria depleted in the Lower NPZ-6 score group (*Ruminococcus obeum* and *Methanosphaera stadtmanae*) previously described as having anti-inflammatory effects (Teichmann and Cockburn, 2021). Consistent with our findings, reduction in butyrate-producing bacteria, such as *Ruminococcaceae*, were reported in HIV-positive individuals with (vs. without) neurocognitive impairment (Zhang et al., 2019; Dong et al., 2021). Also, higher abundances of *Clostridium* spp., a neurotoxin-producing bacteria (Yang and Chiu, 2017) were described in PWH with distal neuropathic pain (Ellis et al., 2022). Aside from studies in HIV, depletion in *Ruminococcus* and positive correlation of this genus with better working memory were reported in individuals with AD and mild cognitive impairment (Liu et al., 2019). Also, *D. desulfuricans* appeared to play a crucial role in the development of Parkinson’s Disease (Murros et al., 2021) as well as *Sutterella* spp. associated with a number of neurological disorders (Hiippala et al., 2016).

The microbial functional profiling identified only one discriminant pathway (propionic acid synthesis (1,2-propanediol degradation pathway) increased in participants with Lower NPZ-6. Interestingly, altered propionic acid levels produced by ASD-associated gut bacteria (i.e., *Clostridium* spp.*, Bacteroides* spp., and *Desulfovibrio* spp) were described as contributing to neuroinflammation (Hiippala et al., 2016; Abdelli et al., 2019). Additionally, propionate, key precursor for lipid biosynthesis, has been found to exert neurotoxic effects through distinct mechanisms, including apoptosis of neuronal cells, increase in oxidative stress, and decrease in glutathione and serotonin levels (Killingsworth et al., 2021).

Cumulative evidence implicates an imbalance of microbiota-derived metabolites in neuroinflammation, contributing to the HAND pathogenesis (Vera et al., 2015; Ouyang et al., 2023). Therefore, we further characterized the host metabolome profiles and found multiple lipid metabolism pathways increased in the Lower NPZ-6 score group and described in other neurocognitive disorders. For instance, ceramide and sphingomyelin have been previously found to accumulate in PWH with progressive neurocognitive impairment (Haughey et al., 2004; Bandaru et al., 2007). Similarly, higher sphingomyelin:cholesterol ratios were associated with poorer performance on memory testing in PWH (Mielke et al., 2010). Increased bile acids and bioactive lipids, along with reduced butyrate-producing bacteria were also reported in HIV-positive individuals with neurocognitive impairment (Dong et al., 2021). Furthermore, other metabolic pathways enriched in the Lower NPZ-6 score group, including sulfur, taurine and nicotinamide metabolism have been associated with neurodegenerative disorders in human and animal models (Zhang et al., 2010; Vitvitsky et al., 2011; Darst et al., 2021).

Through the integrated analysis of microbiome and metabolome data, we found key microbial (*D. desulfuricans, S. thermophilus, S. wadsworthensis* and the 1,2-propanediol degradation pathway) and metabolic features showing correlations and increased in participants with neurocognitive impairment. Interestingly, a previous study described the implication of *D. desulfuricans* in 1,2-propanediol degradation (Ouattara et al., 1992), both displaying the strongest correlations in our results (r > 0.7). Of note, *D. desulfuricans* is a known producer of hydrogen sulfide (H_2_S) in the gut (Karnachuk et al., 2021), acting as a critical signaling molecule in the gut–brain axis via several pathways, including regulating inflammation, oxidative stress, and immune responses, with significant implications for neurodegenerative diseases (Singh et al., 2023) (sulfur metabolism was increased in participants with Lower NPZ-6 score in this study). Furthermore, an imbalance in mucus-degrading microbes, including *Sutterella* spp, has been reported to induce alterations of the mucosal immunity and intestinal epithelial integrity, thereby contributing to immune activation in neurocognitive disorders (Wang et al., 2013).

Such microbial features correlated with metabolites, mostly involved in phospholipid and sphingolipid pathways. Indeed, dysregulation of lipid mediator metabolism, such as ceramide and sphingolipid-derived metabolites, has been linked to the initiation and progression of neurological disorders, including HAND (Liu et al., 2021; Chiurchiù et al., 2022).

By collapsing the discriminant microbial and metabolome features into simple yet intuitive indices, we were able to discriminate between participants with Lower and Higher NPZ-6 score at the study entry, before any intervention. However, such differences tended to lessen over the trial after RMD administration, showing similar patterns between the two group at the study end. We speculate that the observed trend could be explained either by a potential beneficial effect of RMD on the brain functioning (although only participants from the Intervention group received RMD), or global health improvement over time during the trial (approximately 15 months). In support to the first hypothesis, the administration of RMD was shown to have an impact on the plasma proteomic profiles, including inflammatory and neurological marker (in particular, the CD33 protein) in individuals receiving the combined treatment in the BCN02 trial (Duran-Castells et al., 2023). However, the specific factors driving to the observed microbial and metabolic signatures associated to neurocognitive impairment at the study entry and following shift over time cannot be elucidated in this study setting.

In addition, despite our preceding observations identified gut bacteria and metabolic features associated to neurocognitive impairment, potential causal mechanisms underlying these interactions remain unknown and unaddressed. Different mechanisms have been suggested to explain how shifts in gut microbiota composition and related byproducts may affect brain functioning in PWH including triggering of a proinflammatory state, modulation of the microbiota-gut-brain axis signaling pathways and regulation of neurotransmitters and neurotoxic products (Hu et al., 2024). Hence, this proof-of-concept study is indented to provide an associative framework for future validation and functional studies, potentially aimed at exploring novel biomarker and improving non-invasive individual stratification in the setting of HAND.

While this exploratory study provides a framework for more targeted research, several limitations should be acknowledged. The small sample size, lack of clinical validation, and unequal sex distribution between groups—with only one female participant, who belonged to the low cognitive performance group—may have influenced the findings. Sex-related differences in microbiome composition and neurocognitive vulnerability are well documented, and although sex was accounted for in the standardization of the NPZ-6 composite score, a potential confounding effect cannot be entirely ruled out. Moreover, gender-related factors were not explicitly assessed in this study, yet they may also play a role in shaping both cognitive vulnerability and gut microbiome profiles through psychosocial and behavioral pathways. In addition, the absence of published studies investigating gut microbiota signatures in HAND using shotgun metagenomic sequencing hindered cross-validation in independent cohorts. Therefore, the results should be interpreted with caution. Future validation studies with larger, more sex- and gender-balanced samples and balanced group classification are needed, using robust predictive models to confirm and expand upon the proposed signatures.

## Conclusions

Our research provides evidence of associations between gut microbiota and plasma metabolome signatures with neurocognitive impairment in PWH. Although further evidence is needed to establish the precise interplay of the microbiota-gut-brain axis in HAND pathogenesis, future validation studies are crucial for the development of adjunctive treatments and monitoring of CNS alterations. Additionally, this study presents an approach integrating multiple clinical and omics assessments (neurocognition, microbiome, and metabolome), which may serve as an analytical framework for future investigations focused on HAND biomarker discovery.

## Data Availability

Publicly available datasets were analyzed in this study. This data can be found here: https://doi.org/10.1186/s40168-022-01247-6 and doi: 10.1097/QAD.0000000000003121.
