# Supplementary material for: Interactions between gut microbiota, plasma metabolome and brain function in the setting of a HIV cure trial
Source: Front Cell Infect Microbiol. 2025 Aug 20;15:1629901. doi: 10.3389/fcimb.2025.1629901 (PMC12405242; doi:10.3389/fcimb.2025.1629901)
Supplement: Supplementary file 1 [file DataSheet1.docx]

**
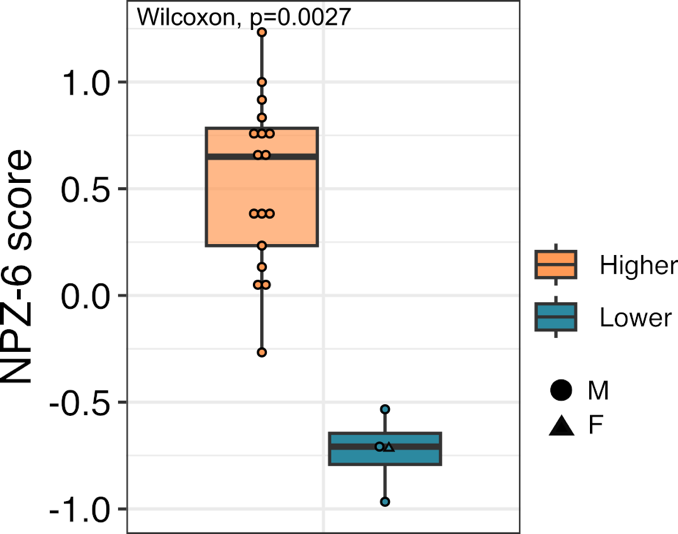
**

**Figure S1.** Comparison between participants with Higher (>-0.5, n=15) and Lower (≤-0.5, n=3) NPZ-6 score at baseline. Male and female participants are indicated with a circle and triangle, respectively.

**
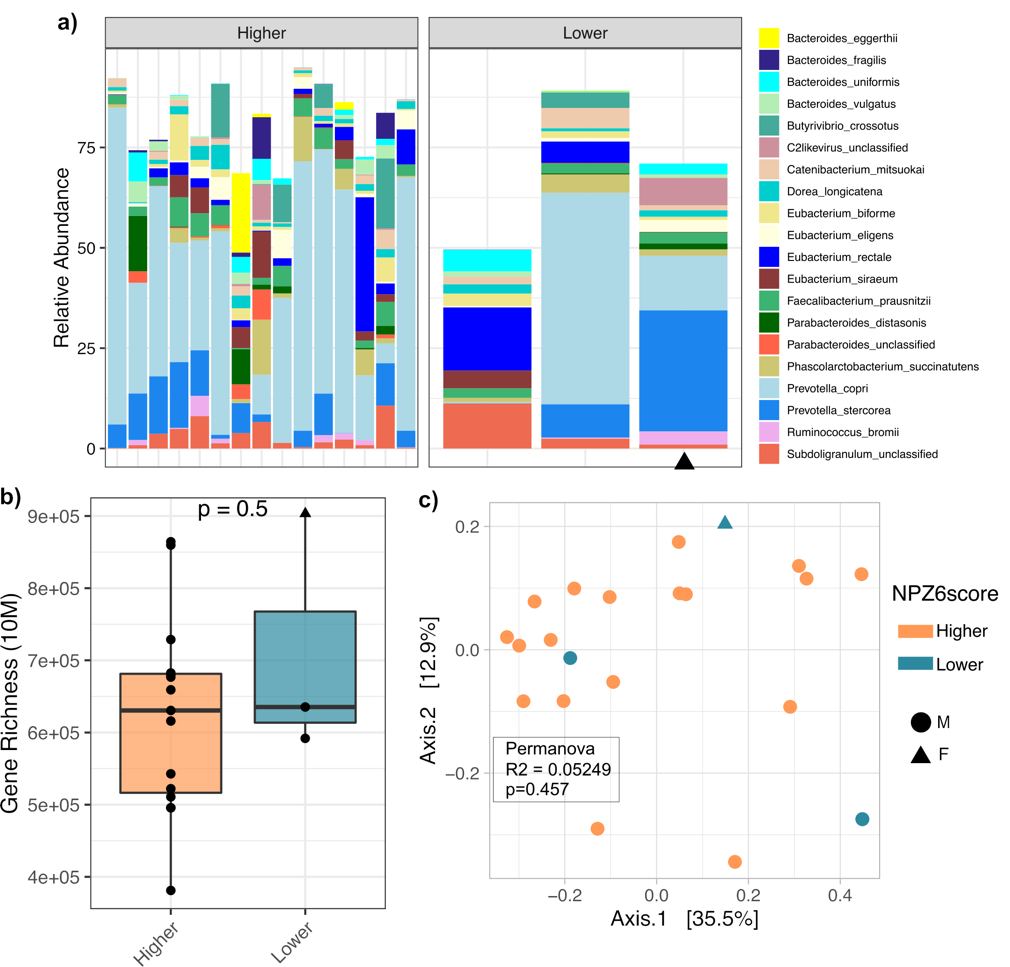
**

**Figure S2.** Species-level composition, gene richness and beta diversity (Bray-Curtis distances) comparison in gut samples from Higher and Lower NPZ-6 participants. Male and female participants are indicated with a circle and triangle, respectively.

**
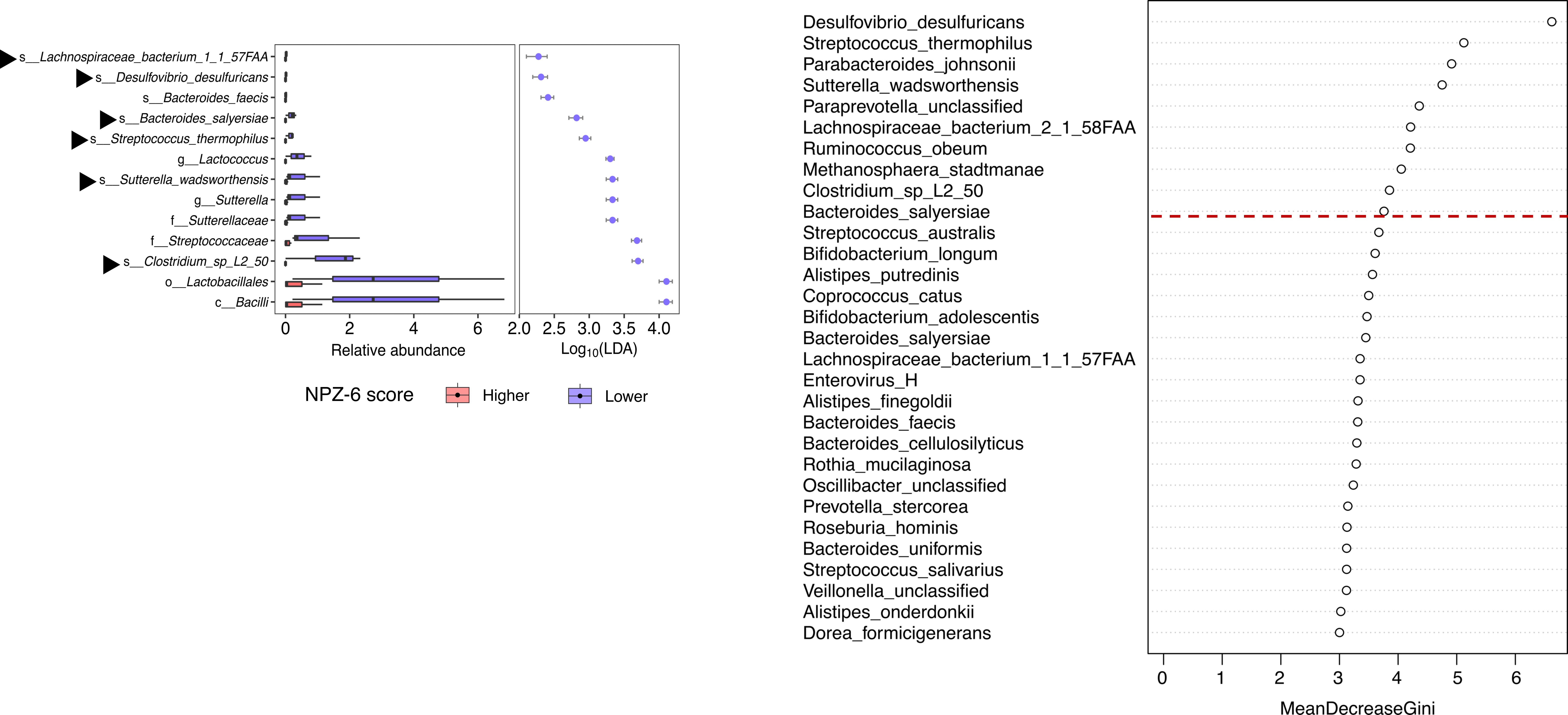
**

**Figure S3.** Random forest validation analysis showing discriminating microbial species between Higher and Lower-NPZ6 groups.

**Figure S4.** Metabolic pathway of L-1,2-propanediol degradation.

**
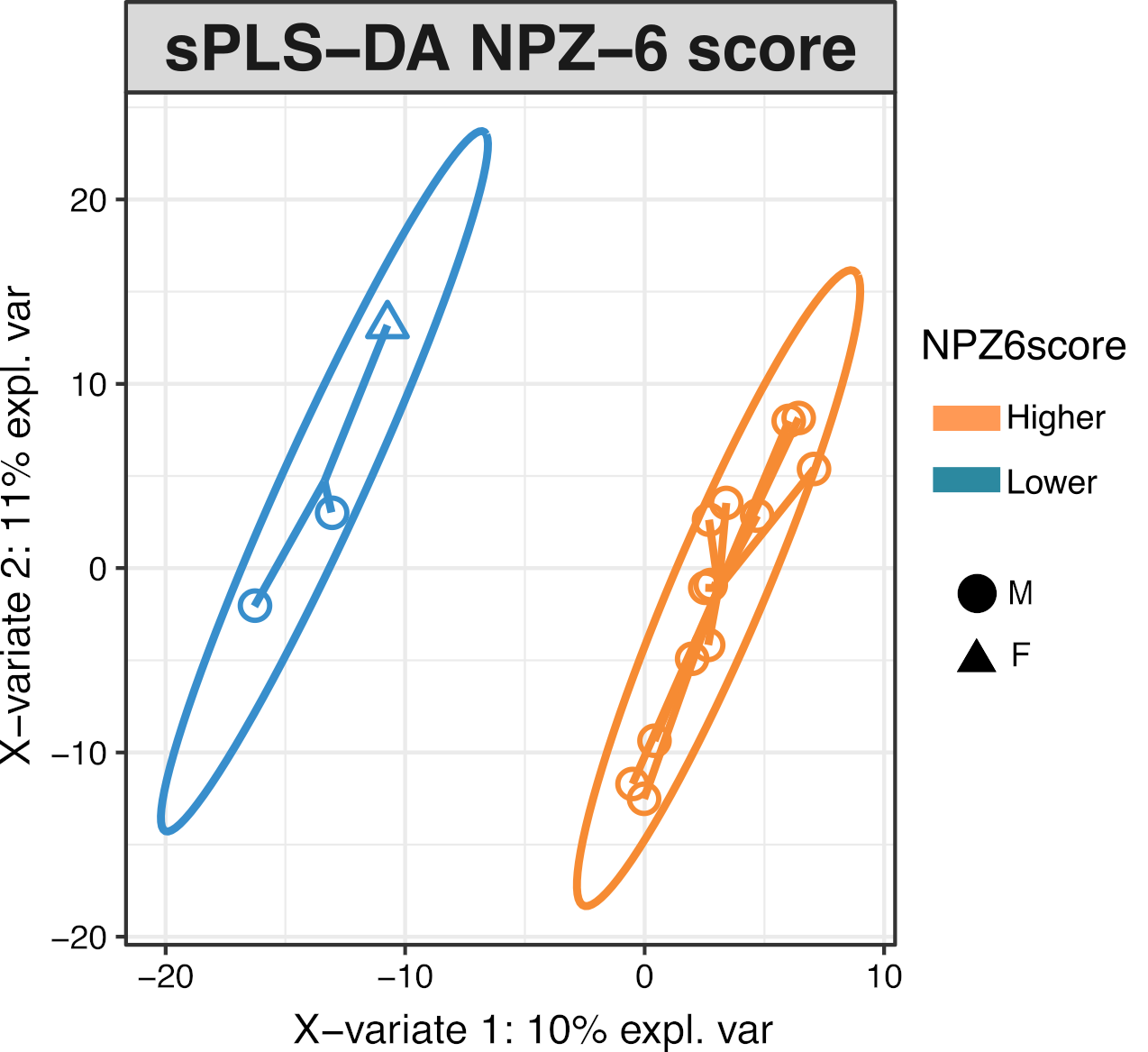
**

**Figure S5.** sPLS-DA sample plot showing discrimination between Higher and Lower NPZ-6 groups based on plasma metabolome data. Top metabolites contributing to separation along components are represented in Figure 2. Male and female participants are indicated with a circle and triangle, respectively.

**
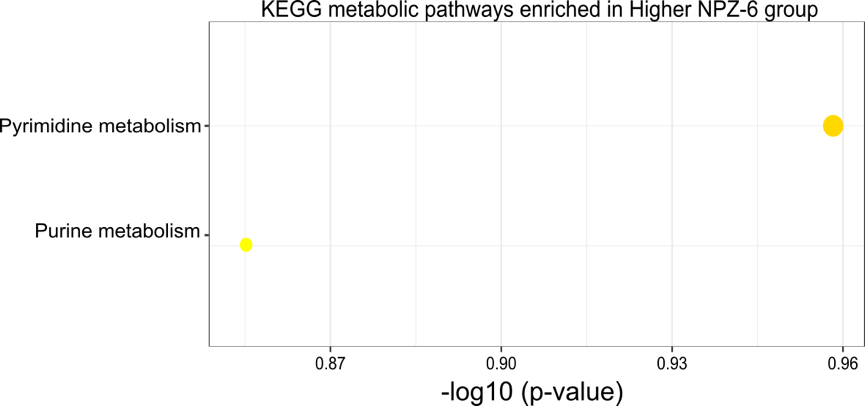
**

**Figure S6.** Enrichment analysis of KEGG pathways in the Higher NPZ6 group using the metabolite sets reported in the differential abundance analysis (Mann–Whitney–Wilcoxon test).

**
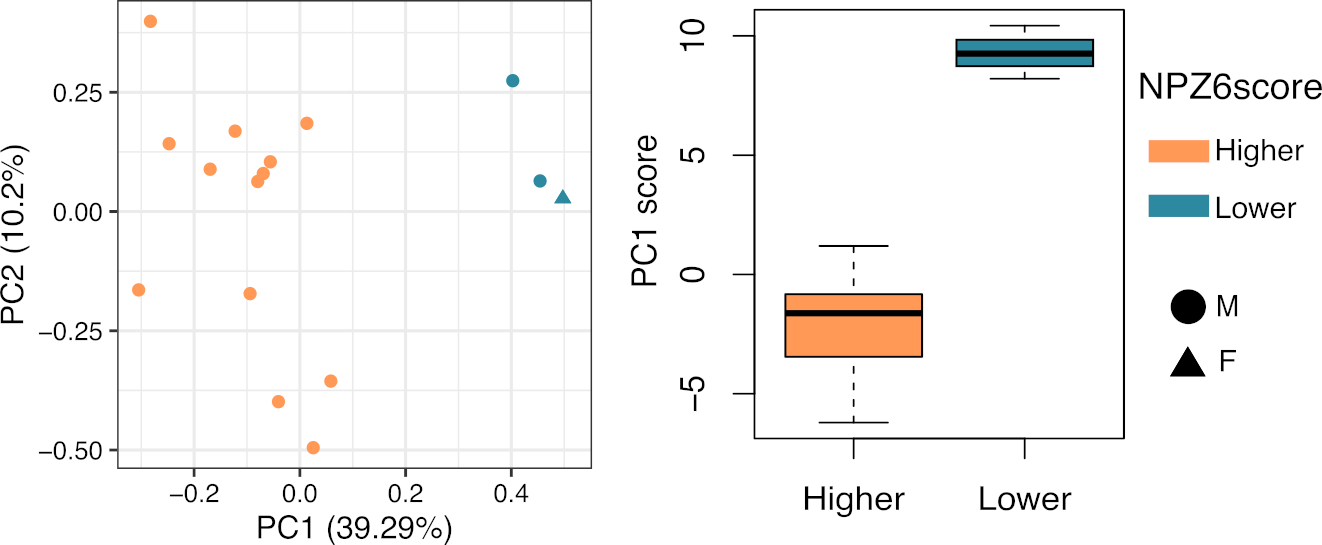
**

**Figure S7.** Principal component analysis (PCA) of metabolites. Male and female participants are indicated with a circle and triangle, respectively.

**
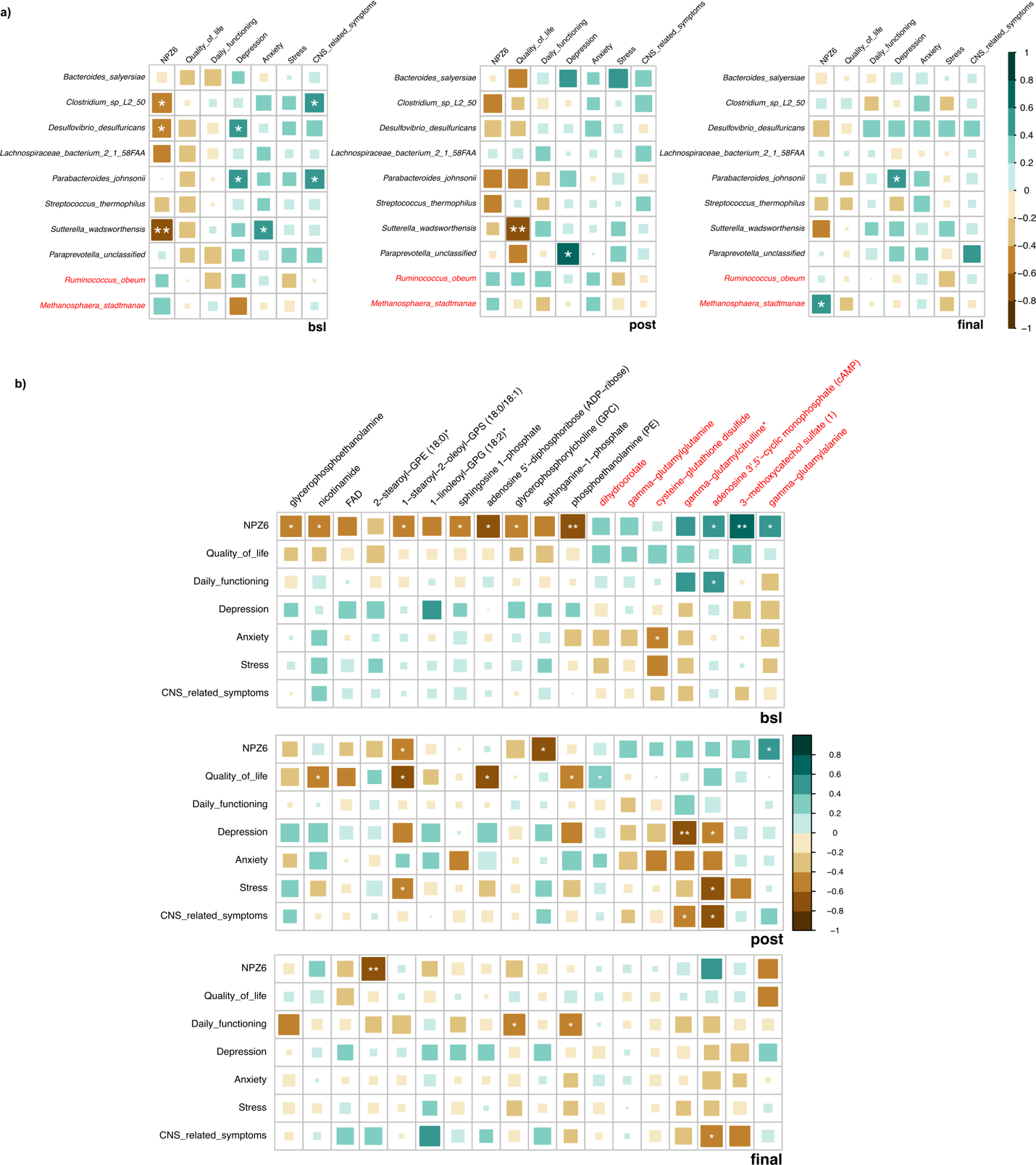
**

**Figure S8.** Heatmap of Spearman's correlations between a) discriminant gut bacterial species and b) metabolites and cognitive and functional outcomes over the trial. Green color indicates a positive correlation while brown color indicates a negative correlation. Asterisks represent correlations with statistical significance (*p < 0.05, **p < 0.01; Benjamini–Hochberg adjustment for multiple comparisons). Features more abundant in Lower and Higher NPZ-6 groups are marked in black and red, respectively.

**
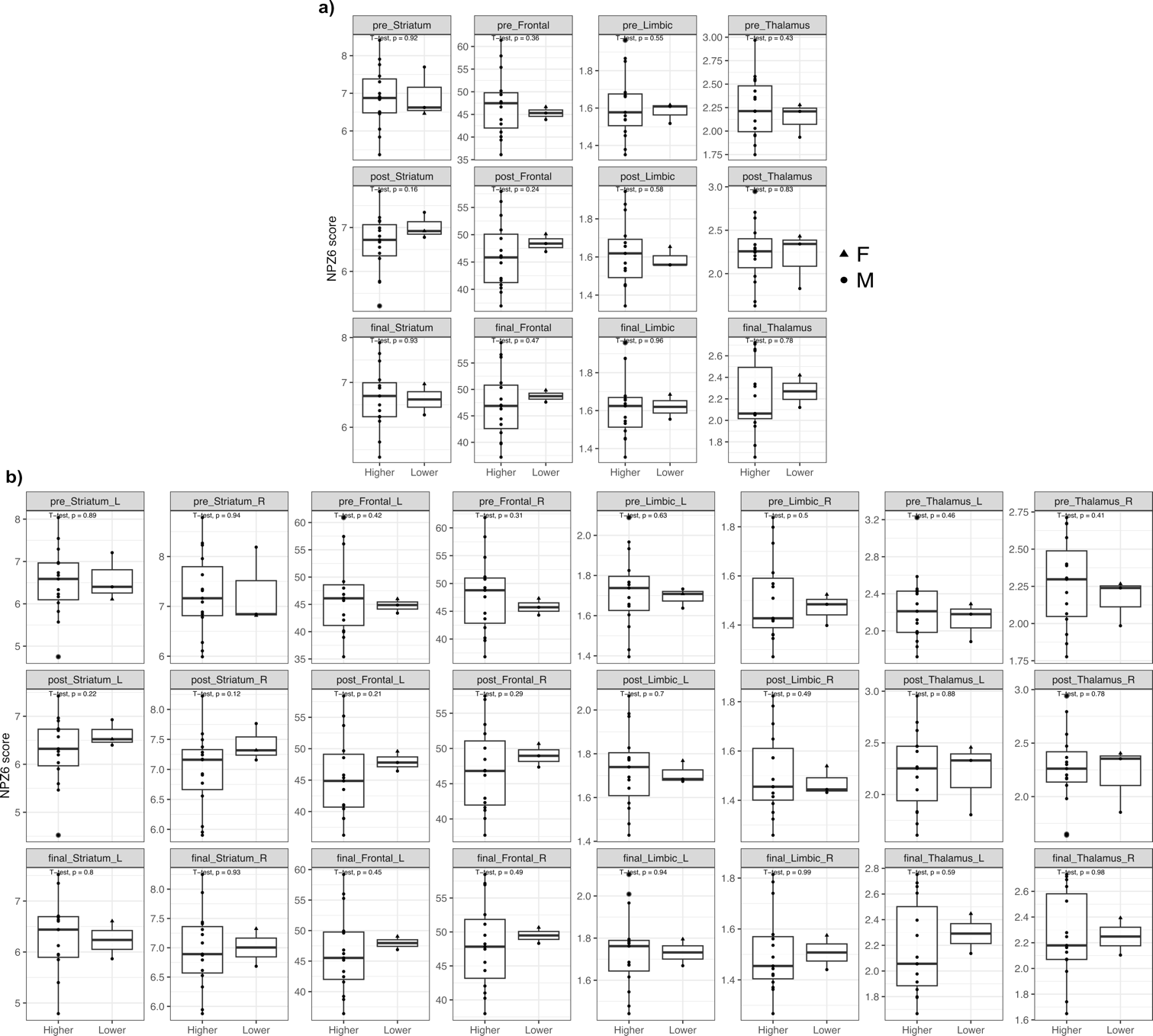
**

**Figure S9.** Comparison of longitudinal neuroimaging measures (voxel-wise volumes in four brain regions) between Higher and Lower NPZ-6 groups at the global level (a) and by brain hemisphere (b). Male and female participants are indicated with a circle and triangle, respectively. *Abbreviations*: L=left, R=right.

**
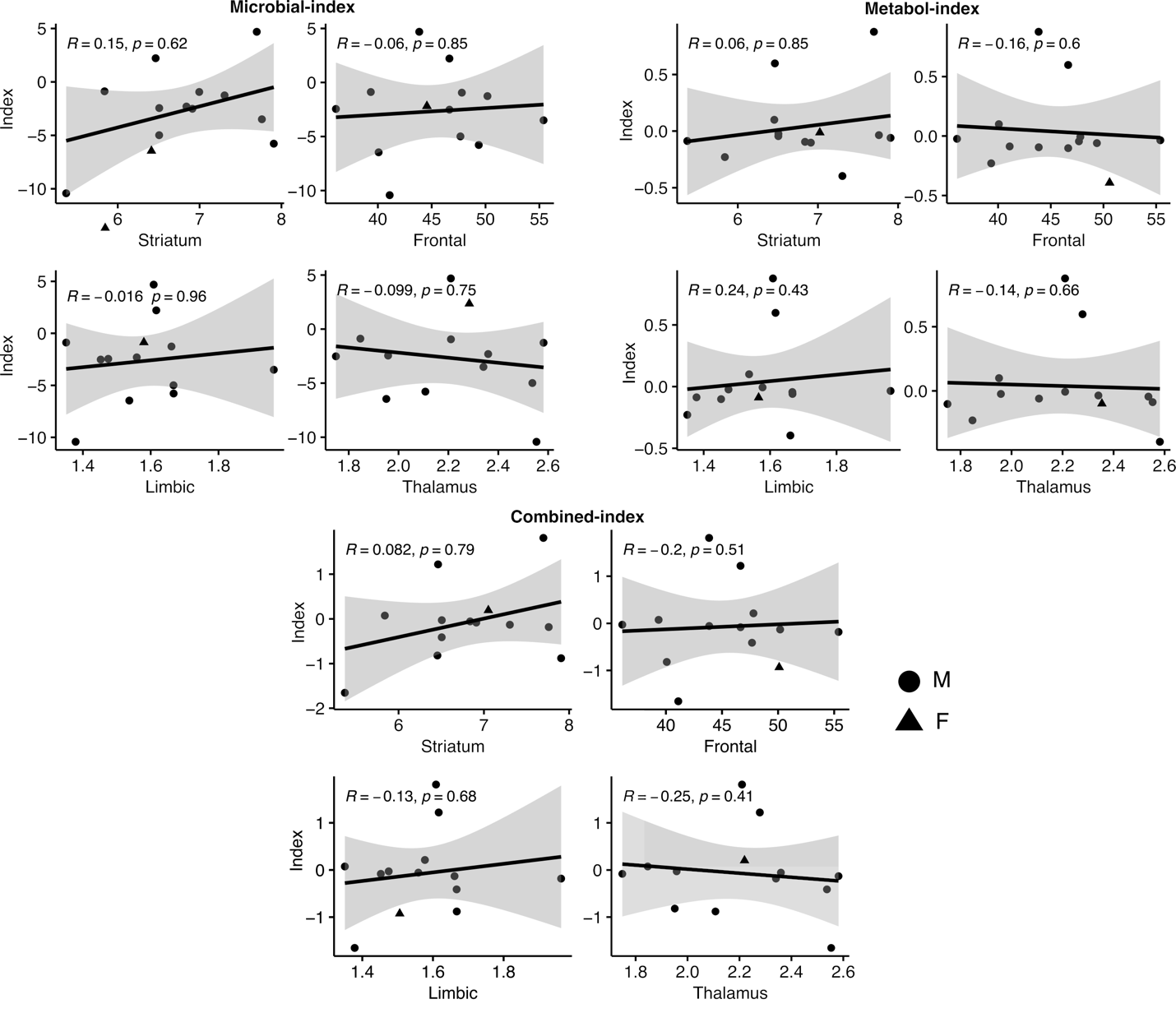
**

**Figure S10.** Spearman’s correlations between predictive indices and neuroimaging measurements at baseline in the four brain regions. Male and female participants are indicated with a circle and triangle, respectively.
